# Interprofessional learning for dental and pharmacy professionals: learning together changes how you work together

**DOI:** 10.1038/s41415-022-4402-8

**Published:** 2022-07-08

**Authors:** Caroline Barraclough, Jalpa Patel, Lesley Grimes, Matthew Shaw

**Affiliations:** 41415129839001 Regional Manager East Midlands, Centre for Pharmacy Postgraduate Education, UK; 41415129839002 Leadership and Transformation Fellow, Health Education England Midlands and East of England, UK; 41415129839003 Head of Learning Development, Centre for Pharmacy Postgraduate Education, UK; 41415129839004 Director, Centre for Pharmacy Postgraduate Education, UK

## Abstract

Can an interprofessionally designed and facilitated learning event change the way professionals understand each other's roles, enable them to better work with each other and improve patient care? Pharmacy and dental professionals are contractors to the NHS, providing services to the public. The way both professions are funded encourages them to generally work in isolation from the wider NHS, in contrast to other areas of healthcare and NHS systems. This study explores how working collaboratively at all stages of design, development, facilitation and engagement of a learning event impacts on the professionals taking part. It also explores how learning interprofessionally can change the way dental and pharmacy professionals work together, suggesting this way of learning is beneficial to improving working relationships between the sectors.

The study explored the ways that shared learning between professions could be approached and encouraged. Pharmacy professionals expressed that they felt more informed and confident giving dental advice to patients. Dental professionals recognised that pharmacy professionals could help support and manage patients. All professionals could see the importance of multidisciplinary working to improve understanding of the other professionals' role. The workshops showed that shared learning is an important aspect to help engage and integrate healthcare systems.

## Key points

- Dental and pharmacy professionals form better working relationships from attending joint interprofessional learning events.
- Addressing topics where there is mutual professional input and expertise promotes better understanding of roles and responsibilities of each professional group.
- A model of interprofessional co-authorship works effectively at producing learning for two healthcare professions, which fosters an opportunity to learn from one another.

## Introduction

Delivering an integrated healthcare system is a key aspect of the NHS Long Term Plan and lack of interprofessional education has been identified as a barrier to promoting this approach.^1^^,^^2^^,^^3^ The development of primary care networks encourages alliance of services between general practitioners (GPs), pharmacists and other community sectors and improved access for patients.^4^ This opens the potential for a multidisciplinary approach to promoting oral healthcare in the community setting.

Primary care dental teams, often working in silo, now have the opportunity to become fully integrated within the healthcare system by tackling oral health issues.^5^ The NHS Long Term Plan outlines intentions to develop the clinical role of pharmacy professionals to become more integrated into the wider NHS.^1^ Building links between disciplines and ensuring a consistent approach to oral healthcare is key to success. This study explores whether these links could be facilitated by learning together.

Pharmacy and dental professionals do not typically learn with other professional groups, nor engage in wider NHS learning activities. From the perspective of the public, however, it seems unlikely that this division is expected; people have an expectation of current practice and evidence-based advice, wherever they access health care.

The main interactions between primary care dental professionals and community pharmacy professionals are related to the prescribing and supply of medicines; typically, when the pharmacy team notes a prescription query or error. If one profession is seen as only pointing out error with the other, trusting and embedded relationships are harder to build, despite clear dependencies on each other.

If considered from the public health perspective, however, the greatest use of both professional groups is a shared desire to maintain a healthy mouth and engage in safe dental treatment. Pharmacies offer a range of equipment aimed at maintaining good oral health and can advise on the impact of medicines on dental practice. Dental teams have expertise in using these products but are not commonly considered to be routine providers.

Community pharmacists see preventive oral health as part of their role.^6^ In 2015, Mann *et al.* investigated the role of community pharmacy in promoting oral health and highlighted that 'community pharmacists can play an important role in oral health promotion' and 'collaboration between pharmacists and other healthcare professionals could offer more effective oral health promotion strategies'.^5^ The study showed that 91.5% indicated they were interested in receiving further training on oral conditions through continuing professional development courses.

Pharmacy and dental professionals have been encouraged to work closer together previously. The Pharmacy Integration Programme was established in 2016 to accelerate the integration of pharmacists across health and care systems.^7^ In 2018, NHS England and NHS Improvement showed their commitment to a more integrated approach to dental healthcare by including specific criteria relating to children's oral health training and assessment in the national community pharmacy quality scheme.^8^ The scheme required 80% of pharmacy staff to complete the Centre for Pharmacy Postgraduate Education (CPPE) children's oral health training and assessment in order to meet the quality criteria.^9^ CPPE developed a gateway page of learning and e-learning on children's oral health for all pharmacy professionals to access.^10^ The aim of this was to engage pharmacy professionals and their teams in the topic of children's oral health, encourage positive oral healthcare interventions in the pharmacy setting and onward referral to dental colleagues. This learning, however, was undertaken as a single professional group (pharmacy only).

The oral health strategy and studies about integration of oral health care into primary care and pharmacy show pharmacists would like more training in oral health care.^2^^,^^5^^,^^11^ They also show there is currently a lack of coordination and integration with other healthcare providers.^2^^,^^7^ This was considered a barrier to collaboration on oral health promotion and informed discussions between Health Education England (HEE) Midlands and East dental colleagues and East Midlands pharmacy professionals from CPPE, to explore integrated training for pharmacy and dental professionals.^9^

Their collaboration led to the development of two joint dental and pharmacy workshops; Workshop A: oral health promotion and Workshop B: anticoagulants, polypharmacy and medication-related osteonecrosis of the Jaw (MRONJ).

The aim of Workshop A was to build links between healthcare professionals, share knowledge, promote a consistent approach to dealing with oral health issues and improve confidence in oral health promotion.

Workshop B, a more advanced clinical workshop, was developed after Workshop A. This was delivered to extend the interdisciplinary learning experience for dental and pharmacy professionals. Both Workshop A and Workshop B were evaluated to establish if learning together on clinical topics would benefit both professions and change practice.

## Aim of this study

The aim of this study was to develop and deliver interprofessional learning to an interprofessional audience and to evaluate the impact of how learning together can change the way pharmacy and dental professionals work together. Through bringing these two professional groups into a shared learning environment, we aimed to explore if perceptions of the two professions could shift, while learning about areas of commonality and shared practice. Could understanding each other's roles and responsibilities better through shared learning events, change the way the professions interacted and worked in practice? This study describes how we undertook this work.

## Method

In both workshops, we developed the learning with an interprofessional team. The workshops were also delivered by a separate interprofessional facilitator team.

### Workshop A

The development of this workshop started with a conversation between HEE in the East Midlands and CPPE about delivering a joint workshop for dentists and pharmacy professionals on oral health promotion. This topic has overlap between the two healthcare settings.

We held an interprofessional consultation evening to ensure the topics and activities engaged both professions. This consultation evening had ten participants in attendance (three dentists, one dental nurse, three community pharmacists, one pharmacy academic and two pharmacy technicians). This was also an opportunity to explore how each profession worked and discuss how they could work together, explain the same principles to patients and ensure patients receive a consistent health message. The consultation evening was designed by the development team consisting of pharmacy, dental and GP professionals.

The consultation highlighted the needs of pharmacy professionals to have resources available to prompt oral health conversations with patients who may seek pharmacy assistance on an unrelated issue. The pharmacy professionals' perspective on how to integrate the promotion of oral health into their already busy service delivery prompted a need for stronger links with dental and GP colleagues to enable appropriate referral to pharmacy. This would facilitate a more integrated approach to oral healthcare in the community. The consultation influenced the design of the programme to include the importance of interprofessional working, local links and better communication between healthcare professionals.

Primary care teams (dental, pharmacy and GP) were invited to the workshop using a combination of online and postal communications. This workshop was open to all members of the pharmacy and dental team and had dental care professionals in attendance. Workshop A was a two-hour face-to-face workshop (ran three times) and included small group learning activities, case studies and presentations delivered by pharmacy and dental professionals. To evaluate the learning, participants were asked to complete a survey before and directly after the workshop.

### Workshop B

A need for a more clinically-focused workshop was identified by a development team of pharmacy and dental professionals. The topics chosen were MRONJ, anticoagulants and polypharmacy. These three topics are important to both disciplines and it was hypothesised both professions could support each other's learning by sharing their perspectives. In particular, MRONJ requires an interdisciplinary approach to its prevention and management. It was felt that both dental and pharmacy professionals would benefit from these topics as they had a common responsibility to patients and overlapping areas of care.

A content verification meeting was held with dental and pharmacy professionals (experts and potential learners) to verify the content was clinically sound and met the needs of both professions. This content verification team comprised of 13 people (five pharmacists, seven dentists and one patient). The workshop followed CPPE's workshop learning style of facilitated, interactive small group learning. CPPE programme design incorporates problem-based learning (Dewey) which allows the adult learner (Knowles) to link the theory to practice and work at their own pace, while learning from each other in small groups. The activities are designed to engage learners of different learning preferences (Kolb) and to encourage reflection and change to current practice (Gibbs). The design of Workshop B incorporates elements of Albert Bandura's social learning theory, to allow learners from both professions to gain information from each other through sharing experiences and learning from observation.^12^The learners were asked to complete an evaluation after the workshop, which focused on whether they felt training with other healthcare professionals impacted their learning and understanding of the clinical topics and how they can apply the knowledge to support patients as part of a wider healthcare team.

### Both workshops

In both workshops, we used an interprofessional development team to develop and verify the content and the workshops were delivered by a pharmacist and dentist working in collaboration. Both workshops included provision of some information on each topic, small group learning and practice-based activities eg case studies.

The interprofessional development team of both workshops ensured the activities not only covered the topic but that the questions specifically focused on how the two professional groups could work together and what insights they could provide, while looking at specific patient examples. Groups were asked to discuss how they would approach the patient scenario so the other profession gained an alternative perspective. This revealed links between what each profession does and stimulated discussion about what would be helpful from the other profession.

The facilitators ensured both professions were heard during the activities and feedback and asked learners to show how each topic or activity could affect their working relationships, as well as improving patient care.

When feedback was taken, the facilitators ensured a balance of perspectives from both professions and joined the feedback together so the learners could see how both professions complemented each other.

## Results

Table 1 shows how many learners attended each workshop, which location they attended and how many of each profession were present. Each workshop was open to 40 learners. Workshop A had an average attendance of 75% and Workshop B's attendance was 55%. The weather conditions on the evening of Workshop B were cited as a barrier to attendance, due to local flooding. Workshop B had 12 pharmacy professionals unable to attend due to the adverse weather conditions. If these pharmacy learners attended, it would have increased attendance to 85%. Average attendance at both workshops was 70% overall.

**Table 1:** Workshop data

Workshop	Date/area	Pharmacy professional	Dental professional	General practice team member	Total number trained/40 places	Percent workshop attendance
Workshop A	Leicester, Sept 2017	23	10	4	37	92.5%
Workshop A	Leicester, March 2018	18	9	0	27	67.5%
Workshop A	Nottingham, Sept 2018	18	5	3	26	65%
	Total number trained in oral health promotion	59	24	7	90	75%
Workshop B	Derby, Nov 2019	5	17	n/a	22	55%
	Total number trained both workshop types	64	41	7	112	70%

There was a different split of attendance between pharmacy and dental professionals for Workshop A and Workshop B. We promoted to both professions and aimed for an even 50:50 attendance to gain balanced representation and discussion from both. In Workshop A, between 62-67% were pharmacy professionals and 27-33% were dental professionals. In Workshop B, 23% were pharmacy professionals and 77% were dental professionals.

Evaluation from both Workshop A and B focused on two broad areas: did they learn about the topic discussed and how did they feel they learned together, networked with other professionals and better understood each other's roles? Tables 2 and 3 outline the written feedback comments from the workshops. Examples of both evaluation questionnaires can be found in Appendix 1 and Appendix 2. Additional quantitative data were gathered for Workshop B.

**Table 2:** Workshop A (where a comment is not attributed to a specific professional group, the profession was not recorded)

Evaluation feedback on topic	Evaluation feedback on learning with other professions
**Knowledge on oral hygiene** Brushing teeth: I have been brushing my teeth incorrectly for 56 years. I now know how to do it, thank you tooth fairies (pharmacy professional) Oral hygiene: Promote oral health to our patients and offer confident advice (pharmacy professional) **Local statistics** Very informative - good speakers Understand oral hygiene better How to save a tooth Best practices when brushing Increase awareness of my patient oral health Give advice confidently on oral health The severity of tooth extraction statistics in Nottingham Promote oral hygiene Be more confident in approaching customers whose child is ready for dental advice (pharmacy professional) Discuss all learning points from tonight at next team meeting Able to offer good teeth-brushing and oral hygiene advice in my pharmacy (pharmacy professional) Pharmacy dental factsheet very useful	**Improved local knowledge and connections** Able to signpost more effectively Make contact with local dental practices for signposting (pharmacy professional) I will contact local dentists and increase communications (pharmacy professional) What we have discussed today I will pass onto my pharmacy colleagues to improve health service (pharmacy professional) Involve receptionists and dental nurses in providing oral health advice (dental professional) Arrange to visit local care homes to provide oral health education to staff and residents (pharmacy professional) **Improved teamwork** Better understanding of how we can work together Involve the whole team- empower the team with knowledge Ask your pharmacy team for advice, be getting good practical advice (dental professional) Interesting to work with dental and doctors, would like more like this (pharmacy professional) **More effective signposting** I will find out where and who to refer to I will signpost to dental services (pharmacy professional) Go and discuss common dental problems with my local pharmacist (dental professional) Identify my local dental points of referral

**Table 3:** Workshop B (where a comment is not attributed to a specific professional group, the comment was shared by both professions)

Evaluation feedback on topic	Evaluation feedback on learning with other professions
15-19 out of 22 felt more confident in their knowledge of MRONJ, anticoagulants and polypharmacy	20 out of 22 felt the workshop increased their confidence working with other healthcare professionals
20 out of 22 said they would train their colleagues on the information learned: MRONJ, polypharmacy and anticoagulants	21 out of 22 felt the workshop increased their understanding of how dental and pharmacy professionals can work together to support patients
**Positive aspects of the workshop**	
Identifying all the different new drugs and being informed about MRONJ Reinforcing up to date guidelines to primary care, hopefully decreasing the increasing referrals Use of British National Formulary app and understanding dental management approaches Covered names/problems of most/all anticoagulants Specific and instructive MRONJ powerpoint and discussion	Working together (multidisciplinary team) Decision making exercises and discussion Discuss case studies Understands dentists work and perspective (pharmacy professional) The interaction, very non-threatening Liked working with pharmacist (dental professional) Interactive nature Fact that we [pharmacist] can be useful to dentist Use of British National Formulary app and understanding dental management approaches
**Least helpful parts of the workshop**	
Discussing the guidelines - I have already had a lot of teaching on this (dental professional) Lots of dental terms I didn't know lots of (pharmacy professional)	Too much group work/more info would be better (dental professional)
**Other comments**	
	Really important we learn to work together and understand how we can work together and share ideas and experiences Cross fertilisation on how the different clinicians interact Useful to liaise with other groups Understand the perspectives of other members

Workshop A's design and initial findings were shared at the University of Manchester Education Conference in 2018.^13^ At the time, the results had not been evaluated. This paper focuses on the qualitative data which offers a richer insight to the perspectives of those present. The evaluation is included here as part of the combined work looking specifically at how dental and pharmacy professionals learning together can affect an integrated approach to oral healthcare in the community.

The evaluation comments show that from both workshops, both professions felt it was important to learn together and that it was helpful to understand each other's roles. Both workshops also had comments about sharing the information learned at the workshops with their extended teams. The evaluation found dental and pharmacy professionals liked learning with each other and both professions were useful to one another in practice.

There were several comments that both professions would like more interprofessional events to learn with dental and pharmacy professionals.

## Discussion

These workshops aimed to move away from the usual interactions between the professions, where pharmacy professionals tell dental professionals when prescription errors have occurred, or changes are needed. They aimed to look forward to a shared approach to patient care and an understanding of roles and responsibilities, to be achieved through learning together in shared topic areas.

There is limited research on the impact of interprofessional learning on postgraduate dental and pharmacy professionals towards their practice and patient care. One study by Sturrock *et al.*, exploring dental professionals' attitudes towards MRONJ and current multidisciplinary team approaches to care, showed that dental professionals felt there was little collaboration between themselves and pharmacists and general communication was around prescribing errors and drug interactions.^14^ The study also showed dental professionals were willing to engage with other healthcare professionals to improve patient care. In particular, better communication with pharmacists was considered to be beneficial to dental professionals.

There are studies on interprofessional learning for undergraduates, particularly medical, nursing and pharmacy, which show an effect on clinical decision-making when students are trained with other healthcare professionals. There is little collaboration between dental and pharmacy undergraduates, at present. Studies of shared learning between pharmacy, medical and nursing students found that students felt that it helped to improve their teamworking skills and would help enhance future professional relationships by better understanding each other's roles.^15^^,^^16^

From the evaluation feedback in this study, each professional group indicated they learned from the topics chosen. From the action planning and next steps activity, they identified one way they will use this information to improve in skill or confidence to affect their practice. Further studies to investigate how practice was changed would need to be undertaken.

The feedback from both workshops was as expected and aimed for. It confirmed the workshops allowed for both professions to work and learn together, to appreciate the roles and responsibilities of one another and explored how they could work better together. There was also a recognition to get in touch with each other in practice after the learning event, showing these workshops were successful.

As we evaluated this work, we reflected on the pharmacy professionals' position after this training when back in practice and if they would be more inclined to make stronger connections and seek perspectives from dental colleagues compared to when they learn as a single profession (pharmacy only). CPPE's children's oral health learning and assessment, undertaken by only pharmacy professionals in 2018, did not enable pharmacy and dental professionals to create better working relationships.^10^ We believe the evaluation comments from both professions in Workshop A and B shows that this example of interprofessional learning increased both professions' ability and desire to work more closely together.

The evaluation was not designed to ask the reasons why people attended each workshop but some reasons could be that pharmacy professionals felt learning about oral health promotion would be more beneficial (Workshop A) than the clinical topics in Workshop B and *vice versa* for dental professionals. Potentially, dental professionals felt more confident in oral health promotion, so had lower attendance at Workshop A. This signalled the need to promote the value of interprofessional networking and cross-professional knowledge sharing to dental professionals to increase attendance at Workshop A and *vice versa* for the pharmacy professionals for Workshop B.

The design of the activities ensured both professions were included in all discussions, specifically about bringing the roles together, which allowed each profession to successfully learn from one another. We believe the importance of developing learning as an interprofessional team enhanced the success of the workshop outcomes.^17^

Despite flooded roads, over 50% of registrants still attended the Workshop B event, which, we believe, shows there is a keen appetite to attend interprofessional learning events.

## Conclusion

There is interest and appetite for pharmacy and dental professionals to learn with, and from, each other. There are clear areas of practice both professions are contributing towards patient care. Neither profession currently receives a full picture, only their siloed interaction with the patient. Learning together aids the overlap of each profession's contribution and improves the patient experience.

This evaluation of two workshops shows that the model of interprofessional co-authorship works effectively at producing learning for two healthcare professions, fostering an opportunity to learn from one another. This is supported by interprofessional co-facilitation at the workshops, to ensure small group learning was truly mixed and that the narrative supported interprofessional working and learning. A limitation to this study, in hindsight, was the reduced detail in information gathering for Workshop A, as the questionnaire did not ask participants to state their profession. If this study was to be repeated in the future, gathering this data could more greatly inform the potential benefits of interprofessional learning for dental and pharmacy professionals. Despite this limitation, this study showed that active learning and small group discussions changed the way that the professions worked together. Through exploring shared issues and exploring patient cases, a working model for the future was developed.

Future research could usefully explore the perceptions of the interprofessional facilitator team on the challenges and benefits of delivering learning in this manner and their recommendations for making interprofessional learning events successful.

**Figure:**
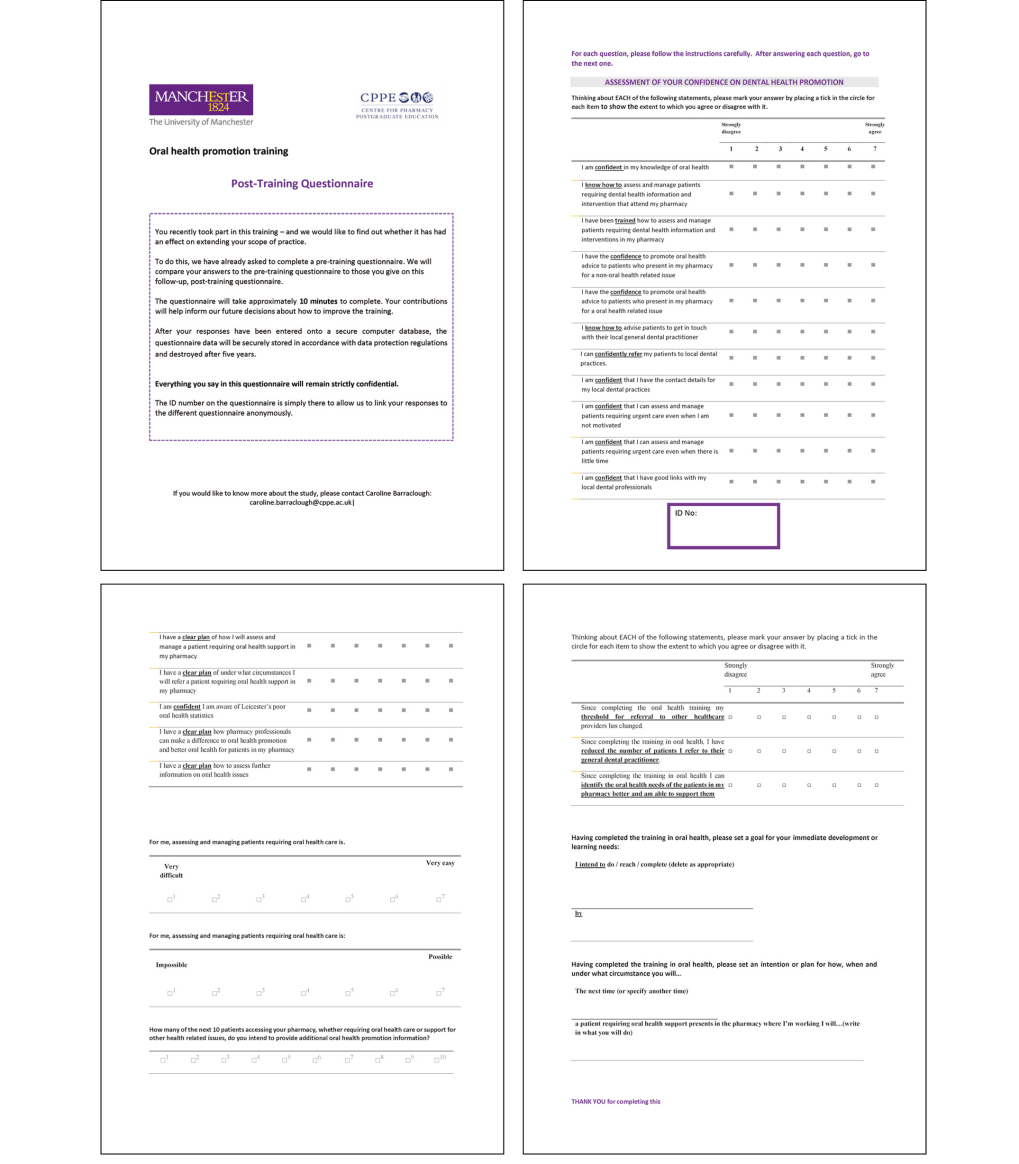
***Appendix 1*** Example of questionnaire handed to Workshop A learners

**Figure:**
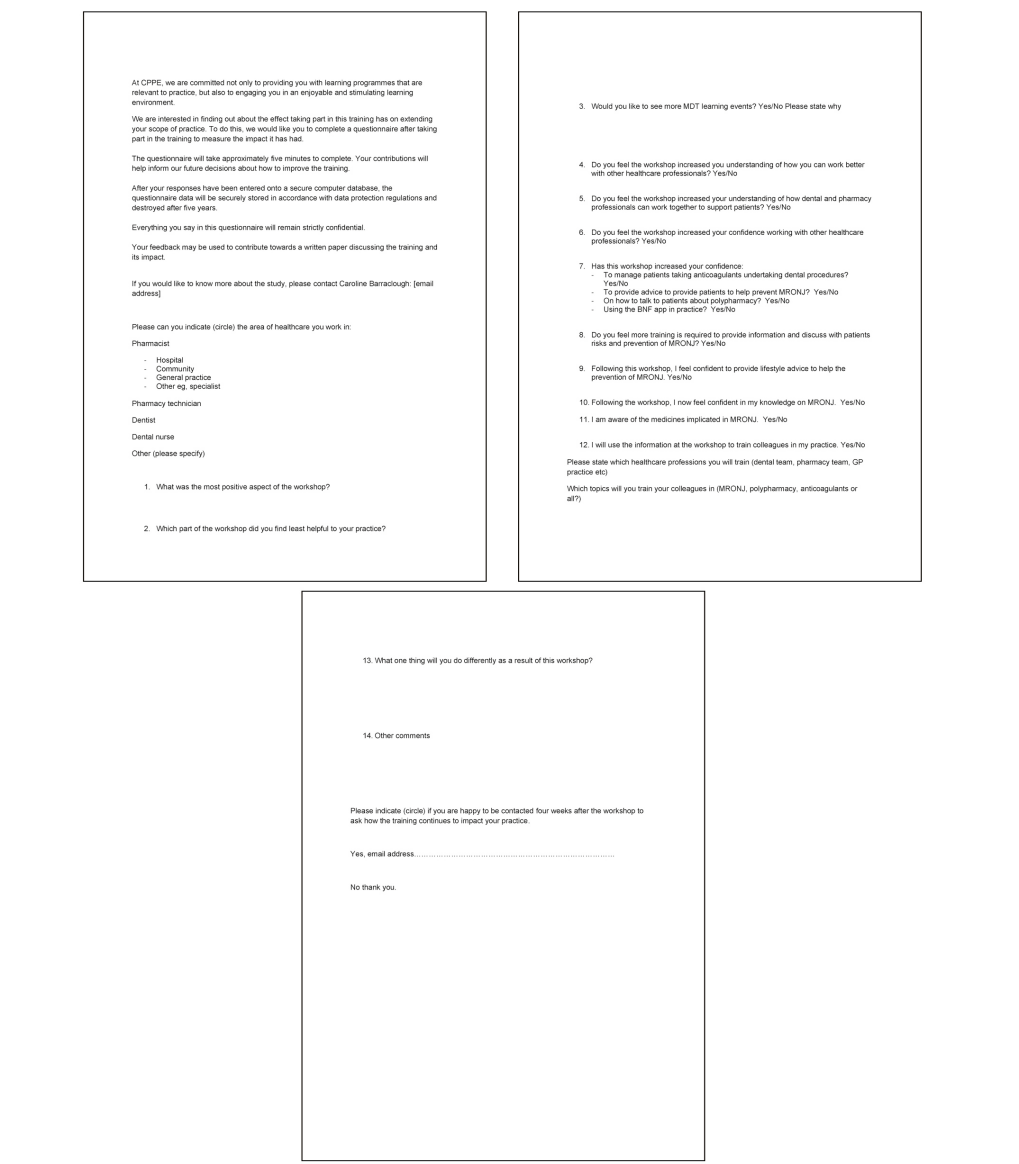
***Appendix 2*** Example of the questionnaire handed to Workshop B learners
